# High Modulus Biodegradable Polyurethanes for Vascular Stents: Evaluation of Accelerated *in vitro* Degradation and Cell Viability of Degradation Products

**DOI:** 10.3389/fbioe.2015.00052

**Published:** 2015-05-06

**Authors:** Melissa Sgarioto, Raju Adhikari, Pathiraja A. Gunatillake, Tim Moore, John Patterson, Marie-Danielle Nagel, François Malherbe

**Affiliations:** ^1^Faculty of Life and Social Sciences, Swinburne University of Technology, Hawthorn, VIC, Australia; ^2^UMR CNRS 7338 Biomécanique et Bioingénierie, Centre de Recherches de Royallieu, Université de Technologie de Compiègne, Compiègne, France; ^3^CSIRO Manufacturing Flagship, Clayton, VIC, Australia; ^4^PolyNovo Biomaterials Pty Ltd., Port Melbourne, VIC, Australia

**Keywords:** polyurethane, properties, degradation, cardiovascular stents, cytotoxicity

## Abstract

We have recently reported the mechanical properties and hydrolytic degradation behavior of a series of NovoSorb™ biodegradable polyurethanes (PUs) prepared by varying the hard segment (HS) weight percentage from 60 to 100. In this study, the *in vitro* degradation behavior of these PUs with and without extracellular matrix (ECM) coating was investigated under accelerated hydrolytic degradation (phosphate buffer saline; PBS/70°C) conditions. The mass loss at different time intervals and the effect of aqueous degradation products on the viability and growth of human umbilical vein endothelial cells (HUVEC) were examined. The results showed that PUs with HS 80% and below completely disintegrated leaving no visual polymer residue at 18 weeks and the degradation medium turned acidic due to the accumulation of products from the soft segment (SS) degradation. As expected the PU with the lowest HS was the fastest to degrade. The accumulated degradation products, when tested undiluted, showed viability of about 40% for HUVEC cells. However, the viability was over 80% when the solution was diluted to 50% and below. The growth of HUVEC cells is similar to but not identical to that observed with tissue culture polystyrene standard (TCPS). The results from this *in vitro* study suggested that the PUs in the series degraded primarily due to the SS degradation and the cell viability of the accumulated acidic degradation products showed poor viability to HUVEC cells when tested undiluted, however particles released to the degradation medium showed cell viability over 80%.

## Introduction

A major driver to develop biodegradable stents is to overcome problems of conventional metallic stents (scarring, thrombosis, and clotting), as well as to eliminate the need to have a permanent implant embedded in the vessel as mechanical reinforcement of the vessel may not be needed once the arterial remodeling and healing have occurred (Heublein et al., 2003; Tsuji et al., 2003). Two of the most promising biodegradable stents that have been tested in human clinical trials are the Igaki-Tamai^®^ and Absorb™ stents. The Igaki-Tamai^®^ stent with a zig zag helical coil design is made from biodegradable poly-l-lactic acid (PLLA) and has been clinically tested (Serruys et al., 2011; Nishio et al., 2012). In the past 3 years, over 60,000 Absorb™ bioresorbable vascular stents have been implanted, and their short-term relative performance against metallic stents have been reported but long-term data is needed before their widespread use (Di Mario and Caiazzo, 2015). Magnesium and its alloys have also been investigated for biodegradable stents but their degradation rate has not been optimized to acceptable levels (Moravej and Mantovani, 2011). Despite these developments, new materials with good biocompatibility, adequate mechanical strength during healing and remodeling, as well as complete degradation of the materials to nontoxic products are still sought after.

Polyurethanes have been used as drug eluting coatings for metallic stents, and there is considerable interest to develop a polyurethane-based biodegradable stent due to their excellent mechanical properties and good biocompatibility, and the wide choice of monomers and formulation options available to tailor polymer structures to meet property and performance specifications of vascular stents (Rechavia et al., 1998).

We previously reported the mechanical properties and degradation behavior of NovoSorb™ series of high strength PUs developed to explore their application in vascular stents (Sgarioto et al., 2014). Evaluation of real-time degradation behavior of these PUs indicated that more than 9 months may be required for complete degradation *in vivo*. In order to better understand the degradation of this PU series and the effect of degradation products on cell viability, an *in vitro* degradation study was conducted at 70°C. Considering the *in vivo* toxicity concerns of PUs in the past (Batich et al., 1989; Guidoin et al., 1992; Benoit, 1993; Williams, 1995), it is essential to investigate the cytotoxicity of biodegradable PUs and their degradation products.

Biodegradable PUs have been investigated extensively for biomedical application, in particular as scaffolds in regenerative medicine therapies, and several studies have reported on the degradation of PUs specifically designed to be biodegradable (de Groot et al., 1997; Dupret et al., 1999; Tuominen et al., 2002; Gunatillake et al., 2006; van Minnen et al., 2006; Yilgor and Yilgor, 2007; Guelcher, 2008). Most of the studies have focused on understanding the rate of degradation under *in vitro* conditions, generally for softer grades of PUs (low HS content). Hafeman et al. (2011) have reported PUs based on lysine diisocyanate and hexamethylene diisocyanate and demonstrated that hydrolytic and macrophage mediated esterolytic and oxidative mechanisms are responsible for poly(ester urethane)s degradation. The primary mode of degradation is hydrolytic where the ester bonds in the SS degrade to respective α-hydroxy acids. The isolation of degradation products due to HS degradation is reported in this paper, but the extent of the degradation and the underlying mechanisms are not clear.

Many of the studies reported in the literature have investigated softer grades of PUs and the series of PUs reported in this study have relatively higher proportion of HS, purposely formulated to achieve high strength for applications such as coronary stents. The study also aims to understand their degradation behavior as well as any toxicity associated with degradation products. Since the degradation is relatively slow in simulated body temperature conditions, we have used accelerated test conditions to force degradation to yield higher concentration of degradation products to assess their toxicity. Although *in vitro* degradation is not a very good predictor of *in vivo* degradation since different mechanisms are operating *in vivo*, this study helps to understand the degradation behavior of harder grades of PUs under hydrolytic conditions and the effect of HS concentration on degradation rate.

The physical changes in the polymer including appearance, and the effect of degradation products on solution pH and cytotoxicity were examined. The effect of HS proportion and ECM coatings on accelerated degradation was also investigated. We also investigated the role of ECM proteins such as collagen (Coll) and fibronectin (Fn) coatings on PU surface to improve endothelialization. This approach has been investigated for other synthetic polymers (Budd et al., 1989; Gosselin et al., 1996; Walluscheck et al., 1996; Feugier et al., 2005; Turner et al., 2006; Nagel et al., 2008) but only few investigations with biodegradable PUs.

## Materials and Methods

### Polymer studies

#### Polymer Preparation

The PU series was developed, synthesized and supplied by PolyNovo Biomaterials Pty Ltd. as previously reported (Sgarioto et al., 2014). Each of the five PUs was synthesized from 1,6-hexane diisocyanate (HDI), isophorone diisocyanate (IPDI) and 1,4-butane diol, while the soft segment (SS) was based a copolymer of l-lactic acid and glycolic acid P(LLA:GA) with MW 1033 with LLA:GA ratio 90:10. The PUs in the series have a HS ranging from 60 to 100% and were labeled as: 60, 70, 80, 90, and 100% HS. The PUs were compression molded into 100 μm thick films using Diamond Fusion^®^ glass plates between 180 and 200°C under a nominal load of 8 tons cut into 1 cm^2^ disks. A digital caliper (Fowler Value-Cal^®^) was used to measure sample thickness and only 100 ± 10 μm thick films were used. Polymer samples were placed in a plastic bag (flushed with nitrogen) and stored in a desiccator at a temperature of approximately 25°C. Polymer samples were sterilized at Steritech Pty Ltd., Vic. and packaged into zip lock plastic bags (25 samples per bag) under nitrogen atmosphere and exposed to 25 kGy of γ-irradiation. The bags were kept sealed in a desiccator at ambient temperature and only opened immediately before their use.

#### Protein Coating

Collagen (Coll, purified bovine Type I collagen, CBPE2, Symatese S.A.S) and Fibronectin (Fn) (human plasma fibronectin, 11051407, Roche Diagnostics Pty Ltd.) were used in this study as a single and double-layered coating. Fibronectin was used at a concentration of 2 μg/mL in PBS (120 ng/cm^2^), Coll was used at 1 mg/mL dissolved in distilled water (60 μg/cm^2^) as per manufacturer’s guidelines. A collagen coating at this concentration has been shown to enable fibril formation *in vitro* (Kreger et al., 2010).

The polymer samples were passively coated following the protocol previously described by Sgarioto et al. (2012) The Fn solution was allowed to adsorb on the TCPS for 45 min, at 37°C and washed with PBS 3 times for 10 min. The Coll was left to adsorb for 10 min at RT and then washed twice in PBS for 10 min. The collagen + fibronectin (Coll + Fn) coating was done similarly except, Coll was coated first followed by the Fn.

#### Accelerated Degradation of Polyurethane Series

Accelerated degradation studies were carried out according to a modified ASTM F 1635 method. Any changes to the solution pH due to accumulating degradation products were not adjusted to maintain solution sterility required for subsequent cell viability studies. Polymer samples (1 cm^2^ disks) were placed in 18 mL glass vials containing 5 mL of 0.1 M PBS at pH 7.4 and incubated in an oven at 70°C for 18 weeks. At pre-defined time points (24 h, 1, 2, 4, 6, 8, 10, 14, 18 weeks), 100 μL of the degradation solution was removed from the test vial and subjected to the toxicity tests. The pH of the PBS was measured weekly over the 18 weeks using a pH meter (Mettler Toledo™) in a concurrent experiment. The samples were removed from 70°C bath and left to cool to RT (minimum of 1 h) before pH measurement. The results represent the mean of three samples ± SE.

#### Physical Changes during Accelerated Degradation

A Gel Doc™ XR system was used to demonstrate the physical change in the polymer during the 18-week period. At pre-determined time points; 0 h, 24 h, 1, 2, 4, 6, 8, 10, 14, and 18 weeks, the bulk polymer sample that had not yet degraded into soluble products was taken from the vial (at 70°C), rinsed in distilled water and any surface water was removed with a Kimwipe^®^. Images of the polymer sample were taken and data acquired using the computer software program QuantityOne^®^ (version 4.6.7) to show the physical and structural changes of the polymer during accelerated degradation. If the sample had degraded into fragments that could not be easily removed, images were obtained of the sample within the storage container.

#### *In vitro* Cytotoxicity Study (MTT Test)

The cell viability assay using MTT (3-(4,5-dimethylthiazol-2-yl)-2, 5-diphenyltetrazolium bromide; Invitrogen™) was undertaken as a measure of the effect of the degrading polymer on cell proliferation and activity. The effect of HS and ECM on cell viability was determined by measuring the uptake and reduction of the tetrazolium salt to an insoluble formazan dye by cellular microsomal enzymes. To reduce experimental error and improve seeding efficiency, the wells that lined the perimeter of the 96-well plate were not used in this experiment. 100 μL of PBS was placed in each of these wells to create a barrier around the seeded wells, ensuring that any evaporation that occurred whilst in the incubator would only occur in the wells containing PBS. The 96-well micro-titration plates were seeded with 10,000 cells/cm^2^ and incubated for 48 h to allow for cell attachment and confluence. After the incubation the medium was removed and replaced with 50 μL of fresh medium except for a designated column one treated as control. 100 μL of degradation solution (not diluted in PBS) was placed into column two and a serial dilution (twofold) of the degradation solution was carried out until column six. After 24 h of incubation, the media containing degradation solution was removed, the wells were washed in PBS and 50 μL of fresh media was added. 5 μL of MTT solution (5 mg/mL PBS) was placed into each well and incubated at 37°C. After 4 h of incubation, 25 μL of the media + MTT solution was removed and 50 μL of dimethyl sulfoxide (DMSO; Sigma-Aldrich^©^) was added to each well for cell permeabilization and incubated at 37°C for 10 min. Absorption at 540 nm was measured using a microplate reader (Biorad Laboratories). The percentage of cell activity after exposure to degradation products was determined by dividing the absorbance of the wells exposed to degradation products and of the control well for that sample. Each sample was performed in triplicate and each experiment was repeated three times. The data represent the mean percentage of cell activity compared to the control well ±SE (*n* = 9). The control well was composed of all components of the assay except the degradation solution.

#### The Toxicity of the Degradation Solution Components

After 18 weeks of accelerated degradation, the individual components of the PBS degradation solution for 60% HS was separated and tested for cell activity. The three components of the degradation solution tested were; the PBS containing degradation particles (complete degradation solution), the degradation particles alone (particles only) and the PBS alone, which is the PBS degradation solution after the degradation particles were removed. The MTT assay was used as a measure of the effect of the separated degradation particles for 60% HS after 18 weeks of degradation on cell proliferation and activity.

Ninety-six well microtitration plates were seeded with 10,000 cells/cm^2^ and incubated for 48 h to allow for cell attachment and confluence. 200 μL of degradation solution was removed from the sample vial of which 100 μL was transferred into a centrifuge tube and subjected to 1,000 RPM for 4 min. The PBS was then removed and the pellet containing degradation particles was re-suspended in 100 μL of media. The 96-well plate was then removed from the 37°C oven and the old medium was replaced with one of the three test solutions; complete degradation solution, PBS only and the degradation particles only. The cells were exposed to 100 μL of each test solution for 24 h at 37°C; test solutions removed and the cells washed in PBS, and 50 μL of media were added to cells in each well. 5 μL of MTT solution (5 mg/mL PBS) was placed into each well and incubated at 37°C. After 4 h incubation, 25 μL were removed and 50 μL of DMSO were added, and incubated at 37°C for 10 min. The absorption at 540 nm was measured using a microplate reader (Biorad Laboratories Inc.). For each experiment, the samples were tested in triplicate and three identical experiments were performed. The data represent the mean cell viability of the test solutions, calculated as a percentage of the total cell viability found with the control well (no products). The control well was composed of media and all components of the assay, except one of the three test solutions which contained PBS.

Data is presented as a digitally enhanced version of the original figure (editing using the exposure option in Photoshop^®^). It was necessary to provide an enhanced version for this figure to correct the photomicrography lack of contrast and to exaggerate the detail of interest.

### Growth curve

Tissue culture polystyrene and the five PUs were seeded with HUVEC and cultured in a humidified incubator (37°C, 5% CO_2_) for 14 days. The endothelial cell growth on each polymer was tested without an ECM coating. The samples were seeded at a density of 10,000 cells/cm^2^. The cells were maintained at 37°C and the polymer samples were collected after 1, 2, 6, and 10 days post-seeding. The samples were then rinsed in PBS to remove nonadherent cells and trypsinized. The proliferation on the coated and uncoated samples was determined by cell counting using trypan blue. The reported data represent the average of three samples.

#### HUVEC Growth on Extracellular Matrix-Coated Polyurethane

Coating studies were carried out on 1 cm^2^ disks, comparing the proliferation on TCPS with the PU series. The polymer samples were coated with each of the ECM proteins and the HUVEC were seeded onto each sample at a density of 20,000 cells/cm^2^ and maintained at 37°C. After 48 h in culture the samples were collected, washed with PBS and a second cell count was performed to determine the proliferation of endothelial cells. This experiment was performed in triplicate and each experiment was repeated twice. The results represent the mean proliferation ± SE of 2 individual experiments (*n* = 6).

#### Visual Analysis of Endothelialization

The growth, structure and endothelialization of the ECM-coated and uncoated polymer surface were examined by fluorescence microscopy. The polymer series was coated with each of the ECM proteins and seeded at a density of 20,000 cells/cm^2^. After 24 h in culture the cells were washed with PBS and fixed with 4% PAF, over-night at 4°C. The cells were then washed with PBS and incubated in the dark at RT in 200 μL of the diluted 2 mM EthD-1 solution (2 μL/mL PBS) and component B of the live/dead stain (Invitrogen™). After 2 h, the stain was replaced with PBS and the cells were gently washed three times. The excess PBS was removed and the polymer sample was transferred onto a glass slide, mounted and viewed under the fluorescence microscope (Nikon Eclipse 50i).

#### Statistical Analysis

The statistical program GraphPad InStat^®^ software was used to analyze the data. Unless otherwise stated, the data were represented as the mean ± SE of nine measurements, corresponding to three samples of coated PU for each ECM protein, with each experiment repeated in triplicate. Comparisons of the *in vitro* cytotoxicity study across the five polymers, four conditions and different concentrations were determined by repeated-measures two-way (mixed model) ANOVA. Comparisons between two parameters, such as Coll-coated 60% HS at 100 and 50% concentration, were assessed by the Bonferroni post-test. Comparison of the toxicity of the individual degradation components was determined by a one-way ANOVA. The Newman-Keuls Multiple Comparison post-test was used to assess the difference in toxicity between two parameters, such as PBS only and particles only. Comparisons between the cell coverage across five HS percentages and four experimental conditions were determined by a two-way ANOVA. A Bonferroni post-test was used to assess the endothelialization of two parameters, such as a Coll-coated 60% HS and Fn-coated 60% HS. The statistical results provided in the figures (when statistical significance was found) show comparisons between the ECM coatings of the same HS percentage only (e.g., Coll-coated 60% HS and Fn-coated 60% HS), not comparison between the ECM coating of different HS percentages (e.g., Coll-coated 60% HS and Fn-coated 70% HS). A *p* value of: <0.05 (*) was considered significant; <0.001 (**) was considered very significant, and <0.0001 (***) was considered extremely significant.

## Results

### Physical changes during accelerated degradation

**Figure 1:**
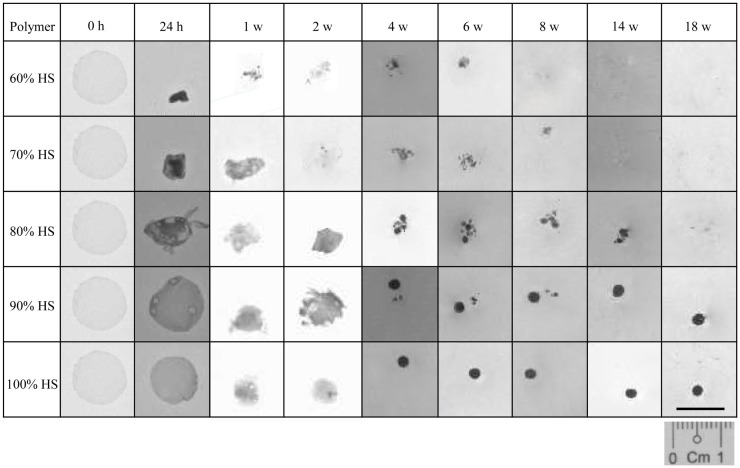
**A comparison of the physical changes to the polymer observed during degradation at 70°C**. Polyurethanes 60, 70 and 80% lost 100% of their original weight after 8, 14 and 18 w, respectively. The weight loss for PU90 and PU100% was negligible.

The percentage weight loss of the degrading polymer was recorded to characterize the physical changes that occurred during 18 weeks of degradation. Figure 1 shows that as the HS increased the effect of degradation on the physical appearance of the polymer decreased. The 60% HS polymer showed the most obvious change during accelerated degradation, with a large reduction in size and structure observed after only 24 h. The small opaque structure that was observed at 24 h eroded into small aggregates at 1 week, which continued to reduce in size gradually over the next 7 weeks until it had completely eroded by Week 8.

The polymers with 70 and 80% HS had similar physical changes during degradation, and identical trends were observed for 90 and 100% HS. The 70 and 80% HS became opaque at 24 h, decreasing gradually in size every week until Week 4, where the polymers changed in structure to form aggregates of spherical particles. The 70 and 80% HS had completely eroded into solution after 14 and 18 weeks, respectively. The samples with 90 and 100% HS became opaque after 24 h but retained the original circular, flat shape despite some reduction is size, presumably due to shrinkage. The polymer samples gradually decreased in size over several weeks, until Week 4, where the polymer samples became spherical, and retained this shape and size until Week 18. The samples that had completely eroded (to the naked eye) after the 18 weeks of accelerated degradation were 60, 70, and 80% HS. The time required for almost complete degradation of 60, 70, and 80% HS was 8, 14, and 18 weeks, respectively. The polymers with 90 and 100% HS underwent only partial degradation, the change in sample size and shape may be a result of shrinkage or softening at 70°C.

### *In vitro* cytotoxicity studies (MTT test)

**Figure 2:**
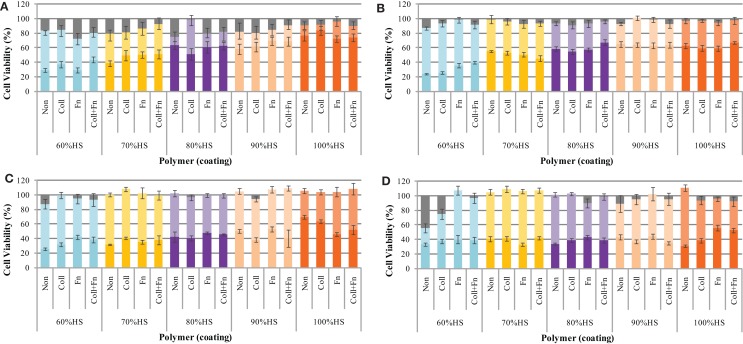
**The cytotoxicity of the degradation solution during accelerated degradation**. The toxicity of the degradation products was determined by a MTT test at (**A**) 1, (**B**) 8, (**C)** 14, and (**D**) 18 weeks of degradation. In each panel the brighter color represents the % cells viability for 100% (undiluted) and lighter color 50% diluted and grey color no degradation solution (control), respectively.

The effect of the degrading polymer during 18 weeks of accelerated degradation on cell viability was determined by a MTT assay. The polymer samples were sterilized, coated with ECM proteins, and placed in oven at 70°C for 18 weeks. At pre-determined time points (24 h, 1, 2, 4, 6, 8, 10, 14, and 18 weeks), 100 μL of the degradation solution was removed and a twofold serial dilution was performed to determine the effect of the eroded polymer fragments on cell viability. A serial dilution was performed to determine the dose-dependent effect of the degradation products on cell viability. The results show that three factors influenced the cytotoxicity: the dose, the HS percentage, and the degradation time (Figure 2).

The cell viability was found to be dependent on the amount of degradation solution that the cells were exposed to. The statistical analysis comparing the five HS percentages and four experimental conditions at each time point (repeated-measures two-way ANOVA) showed that the ECM coating, HS percentage, concentration of degradation products, and the interaction between these factors had a significant effect on cell viability (all results were *p* < 0.05 or lower at each degradation time point).

The cell viability at exposures to 25, 12.5, 6.3, 3.1, and 1.6% degradation solution was very similar to the 50% cell viability; therefore, these values were not shown on the graph. The cell viability of HUVEC after exposure to 50% of the degradation solution was similar to that of 1.6% of the degradation solution. The results shown in Figure 2 only represent the average cell viability after exposure to 100% of the degradation solution, 50% degradation solution, and no degradation solution. At exposure to 50% of the degradation solution, cell viability remained between 100 and 80% for the duration of 18 weeks. The effect of accelerated degradation on cell viability upon exposure to 100% of the degradation products was dependent on the HS. As the HS percentage increased, the effect of degradation on cell viability was also increased. This result was observed at each time point during the 18-week degradation period. In general, the degradation time also affected cell viability. For each HS percentage increase the degradation solution after 24 h was less toxic than the one after 18 weeks. For each PU, the cell viability of HUVEC decreased with degradation time. There was no significant effect of having ECM coating on cell viability, presumably due to active sites on the coating being blocked as a result of strong interaction with PU surface.

### The toxicity of the degradation solution solid components

**Figure 3:**
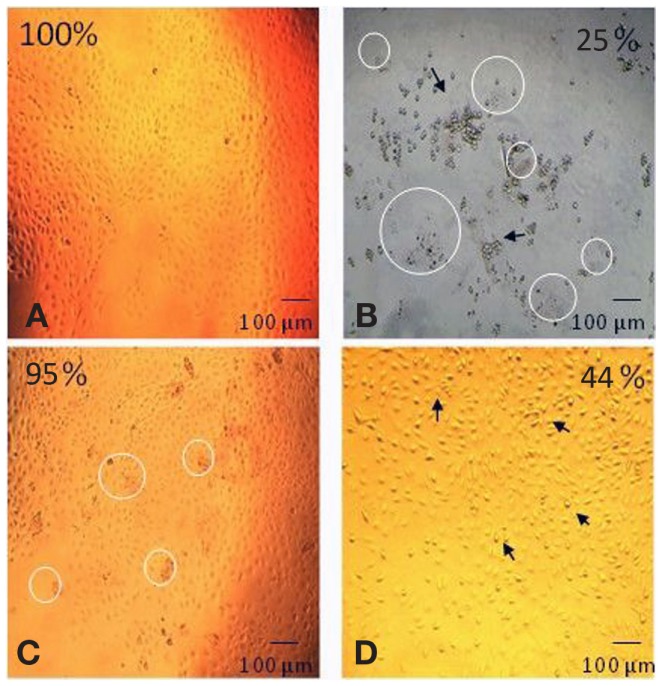
**Digitally altered representation of the cytotoxicity of individual degradation components in the degradation solution**. The degradation solution for 60% HS was separated and tested for cytotoxicity. The morphological changes (and corresponding cell viability) after exposure to (**A**) no products (100%), (**B**) the complete degradation solution (25%), (**C**) particles only (95%) and (**D**) PBS only (44%). The cell viability for the complete degradation solution (particles + PBS) and PBS alone (PBS) was statistically lower (=*p* < 0.001) than the particles alone (particles) and the control (no products). The appearance of degradation particles is highlighted by the *circle* and the *arrow* identifies the apoptotic cells (Bar = 100 μm).

The effect of each component of the degradation solution on cell viability was determined for the 60% HS sample only. The degradation solution was centrifuged to isolate the solid particles from the PBS degradation solution. The cell viability after exposure to the complete degradation solution (particles + PBS), the PBS alone and the degradation particles alone was determined using a MTT test. Both quantitative (MTT test) and qualitative analysis (visual analysis) methods were used to demonstrate and/or investigate the effect of each degradation component (Figure 3) on cell viability.

As shown in Figure 3, the morphology of the HUVEC cells (form and structure) varied depending on the degradation solution component that the cells were exposed to. The HUVEC appearance in the control (no products) was that of a typical endothelial culture, exhibiting a cobblestone arrangement, as evidenced in Figure 3A. This cell morphology was also observed after exposure to the degradation particles only: there were no signs of apoptosis in this live culture (Figure 3C). After exposure to the complete degradation solution, the HUVEC cells formed rounded structures, characteristic of apoptotic cells. The cells completely detached from the TCPS surface and formed several clusters of apoptotic cell aggregates (Figure 3B). Apoptotic cells were also observed in the PBS only sample. The HUVEC in this condition changed in appearance and conformation; from the characteristically flat and spread structure of endothelial cells, to a raised and rounded form (Figure 3D). As highlighted in Figure 3, there was evidence of degradation particles in the cell culture of both the complete degradation solution (Figure 3B) and the degradation particles only sample (Figure 3C).

Figure 3 also shows that the cell viability recorded after exposure to the complete degradation solution was 25 ± 1%, after exposure to PBS alone was 44 ± 1% and for particles alone the viability was 95 ± 4%. The cell viability after exposure to only the degradation particles was statistically similar to the cell viability after exposure to the nondegradation solution (no products). The cell viability after exposure to complete degradation solution and PBS alone was significantly lower than all other conditions (*p* < 0.001 one-way ANOVA).

### pH changes during accelerated degradation

**Figure 4:**
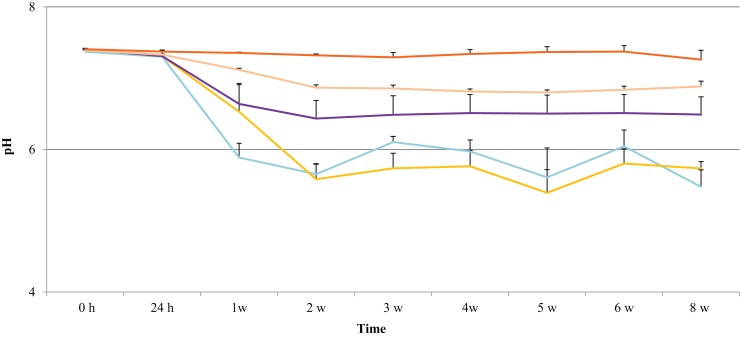
**pH changes in the degradation solution during 8 weeks of accelerated degradation: 60% HS 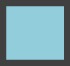, 70% HS, 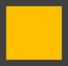, 80% HS 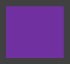, 90% HS 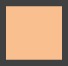, and 100% HS (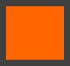, top trace)**.

The pH of the degradation solution was monitored to determine whether polymer erosion had an effect on acidity. The results show that the effect of polymer degradation on pH was dependent on the HS percentage. As shown in Figure 4, the pH for 60, 70, 80, and 90% HS was neutral (7.4 ± 0.2) until Week 1, then the pH decreased to 5.9 ± 0.0, 6.5 ± 0.4, 6.6 ± 0.3, 7.1 ± 0.0, respectively. The decrease in pH for these polymers continued until Week 8, with a respective final pH of 5.5 ± 0.2, 5.7 ± 0.1, 6.5 ± 0.3, 6.9 ± 0.1. The pH for 100% HS remained neutral until Week 8, ranging between 7.4 ± 0.0 and 7.3 ± 0.1.

### Growth curve

**Figure 5:**
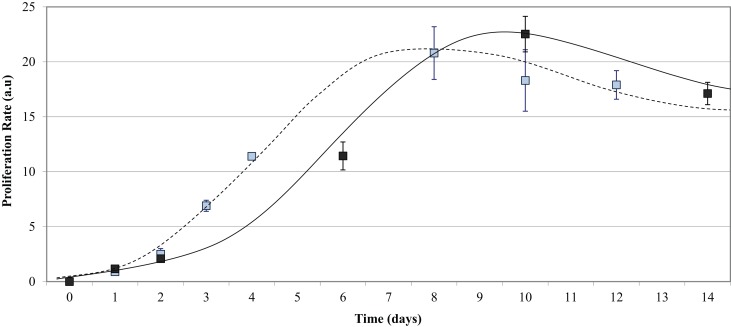
**Endothelial cell growth on the PU series over time**. Compared to TCPS (- - - - blue data points), the PU series (——— black data points) required a longer time in culture to reach endothelialization.

HUVEC proliferation on uncoated PU samples was measured over a 14-day incubation period and compared to the growth on TCPS. The endothelial cells adhered onto all five PU surfaces as early as 1 h post-seeding and were observed as mostly spherical cells. At 48 h post-seeding, the cells had flattened and spread across the surface. Figure 5 shows that the growth of endothelial cells on the polymer series exhibited the general growth pattern of endothelial cells; the proliferation phase was observed between 2 and 8 days, the stationary phase between 8 and 11 days, and the log phase after 13 days. The proliferation on the PU series showed a peak of 23.0 ± 1.6 at Day 10 and decreased to 17.0 ± 1.0 after Day 14 in culture. The endothelial cell growth and proliferation were similar on both TCPS and PU surfaces; however, endothelialization was delayed on the PU. The endothelial cell adherence at 24 h and 48 h was similar on both polymers. A difference in proliferation was demonstrated after 4 days in culture, with a proliferation rate of around 11 on TCPS, which was not observed on the PU series until Day 6. The proliferation on TCPS reached a peak at Day 8, compared to Day 10 on the polymer series. By Day 9, the proliferation on the TCPS began to decrease, whereas the proliferation on the PU series was still within the stationary phase. A final proliferation rate of around 17 was observed for both TCPS and the PU series at Day 12 and Day 14, respectively. This result shows that HUVEC growth on the degradable PU series is similar, but not identical, to that observed with TCPS.

### HUVEC growth on extracellular matrix-coated polyurethane

**Figure 6:**
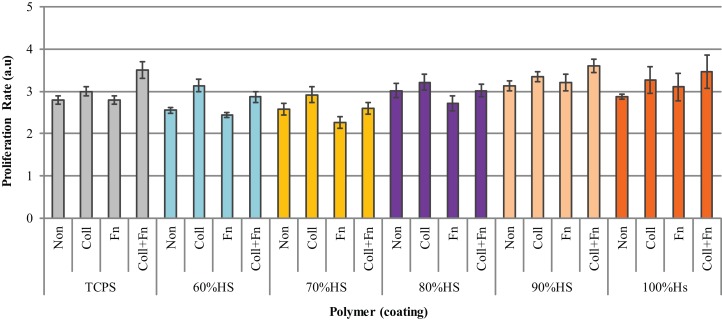
**The difference in HUVEC proliferation on collagen (Coll), fibronectin (Fn), collagen + fibronectin (Coll + Fn) and noncoated (Non) polymer surfaces of all PUs in the series along with results for TCPS**.

Cultured HUVEC were seeded on different ECM proteins at a density of 20,000 cells/cm^2^, and the proliferation rate was measured 48 h post-seeding. Figure 6 shows that the effect of an ECM coating on proliferation was dependent on the type of protein and the HS percentage of the polymer. The statistical analysis comparing the five HS percentages and four experimental conditions (two-way ANOVA) showed that the coating and HS percentage had a strongly significant effect on proliferation (*p* < 0.0001), however, the interaction between the HS and coating was not significant. The statistical analysis comparing the effect of an ECM coating on each HS percentage individually (Bonferroni post-test) showed ECM statistical significance with 60% HS samples only.

As shown in Figure 6, the HUVEC proliferation on Coll was significantly higher than on a Fn coating (*p* < 0.05). The highest HUVEC proliferation observed on TCPS, 90 and 100% HS was on the Coll + Fn coating, with the respective values being 3.5 ± 0, 3.6 ± 0 and 3.5 ± 1. The highest proliferation observed on 60, 70, and 80% HS was on a Coll coating, with the respective values being 3.1 ± 0.0, 2.9 ± 0.1 and 3.2 ± 0.2. For each HS percentage, the endothelial cell proliferation was similar on the uncoated and Fn-coated material, and the endothelial cell proliferation on Coll and Coll + Fn was also similar. In general, HUVEC prefers to grow on a Coll or Coll + Fn coating, when compared to a Fn or uncoated surface.

### Visual analysis of HUVEC on the polyurethane surface

**Figure 7:**
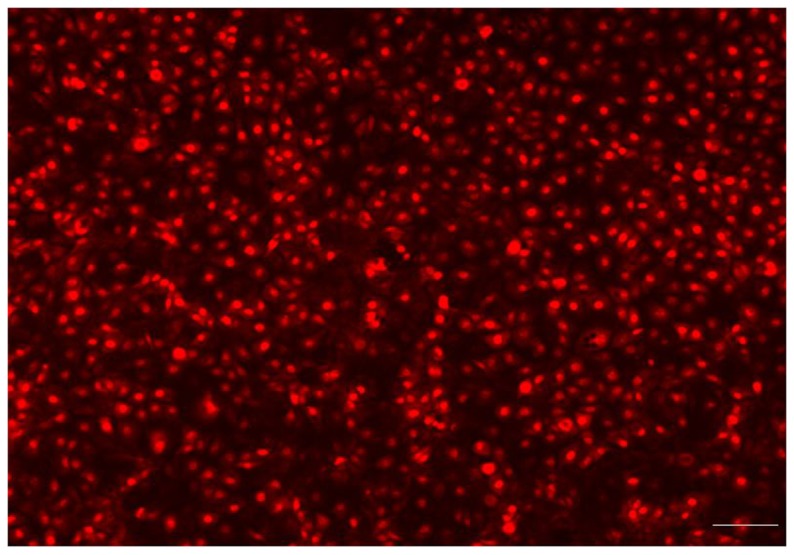
**Fluorescence microscopy showed that the endothelial cells had attached, spread and produced a monolayer with a typical “cobblestone” appearance (Bar = 100 μm)**. The endothelialized PU surface (80% HS) 48 h post-seeding.

To provide evidence that a complete endothelialization occurred on the surface of the PU samples, immunofluorescent microscopy was used. Endothelial attachment was observed on each 1 cm round sample, after 2 h in culture, at a seeding density of 20,000 cells/cm^2^ and complete surface endothelialization was achieved at 48 h. As shown in Figure 7, the HUVEC exhibited a cobblestone appearance typical of endothelial cells on the PU surface. The PU with 80% HS is shown in the figure, the HUVEC morphology was similar on all samples. After 48 h in culture, the cells were flat with a round shape and large nuclei.

## Discussion

Our previous study demonstrated that the PUs in this series were very slow to degrade, over a 9-month period under real-time (PBS at 37°C) conditions (Sgarioto et al., 2014). The present accelerated degradation study was undertaken to force degradation by increasing the temperature to 70°C to understand the effect of HS content on degradation rate and to evaluate toxicity of degradation products as well as the residual polymer (van Minnen et al., 2006). The degradation medium chosen was hydrolytic in nature and assumed that the higher temperature may only accelerate the degradation without altering the degradation mechanism.

Considering the chemical composition of the PUs in the series, a complete degradation of urethane and ester functional groups would yield 1,6-hexanediamine, isophorone diamine, 1,4-butanediol from HS degradation and lactic acid and glycolic acid from the SS. However, due to the relatively slow rate of urethane degradation, the degradation products may be largely associated with the SS degradation. It is also equally important to understand any toxicity associated with the residual polymer fragments, low molecular weight oligomers which are not soluble and retained within the sample.

The results of this study are consistent with those reported in the literature (Tatai et al., 2007) confirming that PUs with lower HS content degrades faster than those with higher HS. Three of the polymers in the series completely disintegrated within 18 weeks of accelerated degradation: 60% HS (8 weeks), 70% HS (14 weeks), and 80% HS (18 weeks). On the other hand, only partial degradation was observed for polymers 90 and 100% HS with no appreciable weight loss. The polymer samples degraded to form small-rounded structures at Week 4, and there was no observable change in the physical appearance from Week 4 to Week 18, presumably due to long HS blocks remaining not degraded.

A good understanding of the biocompatibility of materials and toxicity of compounds generated during degradation is essential before considering any new material for biomedical applications (Gunatillake and Adhikari, 2003; Williams, 2008). As the material degrades not only the physical properties but also the chemical composition, surface properties and morphology undergo significant changes. Understanding how the surrounding tissues react to these changes is critical for the safety of using such materials in clinical applications. *In vitro* studies are a first step in this process and provide valuable information before conducting detailed *in vivo* testing.

As shown in Figure 2, the effect of the degradation products on cell viability when tested in PBS undiluted was highest for the fast degrading 60% HS and decreased with increasing HS content. This tendency was maintained up to 8 weeks and the difference became less significant after that, and in 18 weeks the cell viability was around 40% for all materials. The cells were exposed to seven different concentrations of degradation products: 100, 50, 25, 12.5, 6.3, 3.1, and 1.6%. As there was no observable difference for concentrations ranging from 1.6 to 50%, values recorded for concentrations below 50% were not displayed on the graph. This apparent lack of dose dependency for concentrations below 50% dilution may be attributed to this range being below the concentration level of toxicity of these components. In slow degrading materials the soluble degradation products will continually be removed from the implant site causing no advertise effect to surrounding tissues. It should be noted that the undiluted solution was tested in PBS while other samples were diluted with media. In most cases, there was a statistically significant difference in cytotoxicity when cells were exposed to 100% of the degradation products compared to 50%.

The cell viability of degradation products diluted to 50% was between 100 and 80% for the duration of 18 weeks for all samples, statistically similar to control PBS solution with no degradation products. As discussed earlier, the major degradation products of these PUs are derived from the hydrolysis of the SS based on lactic and glycolic acids, and this is supported by the observation that pH decreased to 7.26 for 100% HS and 5.48 for 60% HS (Figure 4) During the accelerated degradation study, the pH of the degradation solution was not maintained at 7.4 (± 0.2) (van Minnen et al., 2006) since the pH could not be measured while maintaining sterility. However, a parallel non-sterile experiment was undertaken under the same experimental conditions to monitor the pH changes. Previous reports (Yeomans et al., 1985) have shown that the normal pH range of umbilical vein blood is 7.35 ± 0.05. Since the cells used in this study were harvested from the umbilical vein it can be assumed that their growth would be optimal at a pH of 7.35 ± 0.05. The pH for 60, 70, 80, and 90% HS decreased below this range after 1 week of accelerated degradation, whereas this result was not observed with 100% HS until Week 3.

These results suggest that the acidity of the degradation solution is the primary reason for observed effect on cell viability of 100% degradation solution, reaching levels to cause apoptosis of HUVEC at 1 week for all PUs, except 100% HS (see Figure 4). No attempt was made to fractionate and identify the compounds and polymer fragments formed during the degradation. To elaborate on this further, when the samples had completely disintegrated into solution (8 weeks for 60% HS, 14 weeks for 70% HS, and 18 weeks for 80% HS), their effect on HUVEC cell viability was the same, around 40%, for all PUs. However, the cytotoxicity varied at other time points presumably due to varying concentrations of degradation products. Similar results have been observed for PUs by other groups. A study by van Minnen et al. (2006) reported the accelerated degradation (60°C) of PU foams in distilled water for 52 weeks, and the degradation solution was collected at different time points. Cytotoxicity was observed after 3–5 weeks in the undiluted degradation solution (100% concentration), and became more cytotoxic toward the end stage of the degradation, due to accumulation of degradation products, consistent with what we have observed in this study. In our study, we estimated the amount of degradation products released to the medium by determining the polymer weight loss; each week approximately 0.3, 0.2, and 0.1 mg/mL, for 60, 70, and 80% HS, respectively released to the degradation solution. These results confirm that erosion of the polymer increases the concentration of degradation products with time; however, under *in vivo* conditions it is unlikely that soluble degradation products are concentrated at implant site as the body can excrete those from the site, except where large volume implants are involved producing high concentration of degradation products.

Most polyester PU degradation studies show that PUs degrade faster in *in vivo* conditions compared to *in vitro* developed to simulate a biological environment (Zhang et al., 2002; Adhikari et al., 2008). Zhang et al. (2002) found the *in vivo* degradation rate of PU for subcutaneous implantation in rats was three times faster than *in vitro*. This study did not undertake *in vivo* analysis on the biodegradation of this polymer series, however, there is literature on the degradation behavior of PUs in different *in vivo* conditions (Bruin et al., 1990; Saad et al., 1997; Adhikari et al., 2008; van Minnen et al., 2008). An *in vivo* PU degradation study by van Minnen et al. (2008) found no observable toxicity issues when PU foams were implanted subcutaneously in rats and rabbits. After 3 years, the results indicated near complete resorption of the PU foam with no observable cytotoxicity caused by either the degrading polymer or its degradation products. During the early stages of degradation, the number of macrophages and giant cells increased, then gradually decreased over time. The implant was completely resorbed, after 3 years, *in vivo*. Although this study did not outline the nature of the degradation products and how they were absorbed/released from the implant site, it is one of only a few studies that have documented the longer-term degradation of a PU implant, demonstrating near complete degradation.

To ascertain whether the residual particulate matter released to the degradation medium contributes to cell viability, the solution from 60% HS degradation after 18 weeks was centrifuged to separate solid degradation particles. The cell viability after exposure to (B) the complete degradation solution was 25 ± 1%, (D) the PBS alone was 44 ± 1% and (C) the degradation particles alone was 95 ± 4% (Figure 3). These results suggest that the observed effect on cell viability after exposure to the degraded solution is not caused by the degradation particles themselves. The large-eroded polymer fragments visible in the degradation solution (Figure 3) do not affect endothelial cell viability when suspended in media. The cell viability was mostly affected by the PBS, which would suggest that the acidic pH of the PBS was the biggest inducer of apoptosis. There is also a 19% significant difference (*p* < 0.001) in the cell viability after exposure to the PBS alone and the original degradation solution with PBS plus degradation products. This difference could have been caused by the presence of soluble fragments produced during the degradation of the SS, which may have caused cytotoxicity. These molecules would have also been evident in the “particle only” sample but it would have had little effect as the number of particles to the volume of media (which the particles were re-suspended in) would have been too high to elicit an effect. This result showed that the large-eroded polymer fragments themselves are nontoxic when diluted in a nutrient rich media suggesting that *in vivo*, the products produced during degradation of this polymer series would be nontoxic to the cells surrounding the cardiovascular stent.

Previous research on the present PU series has also shown that the materials surface has an excellent ability to support endothelial cell growth and retention (Sgarioto et al., 2014). There are also many other *in vitro* cell attachment, proliferation, and cytotoxicity studies that have shown that biodegradable PUs with a wide range of chemical compositions, have acceptable cytocompatibility. Several studies have reported endothelial cells (Yeomans et al., 1985; Guan et al., 2002, 2004; Zhang et al., 2002; Gunatillake and Adhikari, 2003; Li, 2007; Tatai et al., 2007; Adhikari et al., 2008; Williams, 2008; Laschke et al., 2009; Li et al., 2009) chondrocytes (Saad et al., 1999; Chia et al., 2006; Karbasi, 2009; Adhikari et al., 2010; Bonakdar et al., 2010) osteoblasts (Wang et al., 2000; Zhang et al., 2000; Bonzani et al., 2007; Hill et al., 2007; Schlickewei et al., 2007; Hafeman et al., 2008), fibroblasts (Ganta et al., 2003; Harris et al., 2006; Guan et al., 2007; Henry et al., 2007; Choi et al., 2008; Craciunescu et al., 2008; Fromstein et al., 2008; Nieponice et al., 2008; Rechichi et al., 2008; Raghunath et al., 2009; Wang et al., 2009; Parrag and Woodhouse, 2010) and stem cells (Fromstein et al., 2008; Nieponice et al., 2008; Li et al., 2009; Raghunath et al., 2009; Wang et al., 2009) attachment, growth and proliferation on different types of biodegradable PUs. To further investigate the growth of endothelial cells on the PUs, the growth curve pattern was compared to TCPS. Tissue culture polystyrene is often used as a control for many polymer and tissue culture studies (Saad et al., 1997; Guan et al., 2002; Ganta et al., 2003; Choi et al., 2008; Greenwood et al., 2010) as it is known that cells grow well on TCPS.

As shown in Figure 5, the cell adherence and proliferation study of the PU series exhibited a similar growth pattern to that of TCPS, except endothelialization of PU was slow, needing 28 h more than TCPS. This difference may be attributed to the difference in surface hydrophobicity; the average PU contact angle was 80° compared to 71.8° for TCPS. Vogler (1999) reports hydrophilic materials better support and encourage cell attachment and growth compared to hydrophobic materials. Our previous study (Sgarioto et al., 2014) on ECM-coated TCPS found that a double coating of Coll + Fn exhibited a significantly higher proliferation and migration compared to a single Coll or Fn coating. In this perspective, the surface of the polymers was coated with three different coatings (Coll, Fn and Coll + Fn) to improve cell attachment and retention. Figure 6 shows that HVUEC proliferation was highest on either a Coll or Fn coating, when compared to other coatings on the same HS percentage. The highest proliferation of endothelial cells was observed on TCPS, 90 and 100% HS with a coating of Coll + Fn, whereas the highest proliferation observed on 60, 70, and 80% HS was on a Coll coating. The only significant differences between protein coating were observed with TCPS and 60% HS (Figure 6). In general, across all HS percentages, an ECM coating did not significantly improve HUVEC proliferation.

It appears from this study, along with previous studies on this PU series that the surface of this polymer series does not require surface modification to maintain cell adhesion. The results showed that the effect of an ECM coating is influenced by the underlying polymer base, and is likely to be related to the surface structure and/or contact angle of the different polymers. The contact angle of the PU series was around 80°, which is considered as hydrophobic compare to TCPS 71.8° which is considered as being hydrophilic (Bahulekar et al., 1998; Vogler, 1999). This difference in hydrophobicity/hydrophilicity will affect the ability of cells to interact and bind to these materials. It will also affect the ability of proteins to adsorb to the surface of both the PU and TCPS. Although some-what speculative, it is possible that since the PU series is hydrophobic, Coll and Fn deposition and adsorption would occur more strongly to the PU surface through hydrogen bonding. This may cause the cell binding sites on the proteins to be immobilized and are thus unavailable for interaction with the HUVEC. In the case of TCPS, the deposition and adsorption of Coll and Fn may be less strong, therefore the cell-binding sites are still available for attachment by HUVEC. The results suggest that differences in HUVEC proliferation on ECM-coated PU compared to the results observed on TCPS is associated with differences in hydrophobicity/hydrophilicity and surface functional groups between the two polymers. It is clear from the results of this study that HUVEC proliferation and growth is influenced by the surface structure of the polymer. The cytocompatibility question that cannot be answered from this investigation is whether or not the by-products from polymer degradation will be toxic to inflammatory cells, in particular phagocytes (including macrophages). Theoretically, the eroded polymer fragments in the arterial wall will be ingested by macrophages and subsequently excreted by the body. If polymer fragments enter the bloodstream they will be ingested by monocytes. The roles of the macrophage/monocyte are both nonspecific and specific phagocytosis of any foreign material when it comes in contact with, including bacteria, cellular debris and synthetic particles (Doshi and Mitragotri, 2010).

In summary, the accelerated study was performed to understand the hydrolytic degradation of high modulus PUs. The results showed that the time required for complete degradation of this PU series was dependent on the HS percentage. Only three polymers completely degraded/disintegrated during the 18 week; 60, 70, and 80% HS. The MTT assay demonstrated that the degradation products when tested undiluted showed an effect on cell activity but when diluted to 50% degradation products and below did not affect HUVEC cell proliferation, suggesting concentration threshold below which no apparent effect. The degradation products were diluted in media (1:2 dilution) and the cell viability was around 80% for the duration of the accelerated degradation study. At 18 weeks, PUs that had completely degraded into solution had a similar effect on HUVEC cell activity, irrespective of their HS percentage. This result suggests that cell viability was not caused by the actual chemical components (oligomers or monomers) produced during degradation, but instead the accumulation of these acidic products in the degradation solution. The results showed that the growth pattern of the PU series was similar to that observed with TCPS showing that these polymers have excellent surface characteristics for HUVEC growth and proliferation. For most of the polymers in the series, the degradation solution became acidic over time affecting HUVEC activity. As the HS decreased, the pH decreased due to accumulation of SS degradation products. The results from this study suggest that the mixture of degradation particles produced during the degradation may not be toxic, however, it is necessary to undertake *in vivo* studies to determine whether or not the body has the ability to buffer the acidity caused during degradation as well as to metabolize and excrete the degradation products.

## Conflict of Interest Statement

The authors declare that the research was conducted in the absence of any commercial or financial relationships that could be construed as a potential conflict of interest.
